# Osteopontin-c mediates the upregulation of androgen responsive genes in LNCaP cells through PI3K/Akt and androgen receptor signaling

**DOI:** 10.3892/ol.2015.2939

**Published:** 2015-02-06

**Authors:** TATIANA MARTINS TILLI, LUCIANA BUENO FERREIRA, ETEL RODRIGUES PEREIRA GIMBA

**Affiliations:** 1 Molecular Carcinogenesis Program, Research Coordination, National Institute of Cancer, Rio de Janeiro 22743-051, Brazil; 2 Institute of Molecular Pathology and Immunology, University of Porto, Porto 4200-465, Portugal; 3 Natural Sciences Department, Health and Humanities Institute, Fluminense Federal University, Rio das Ostras, Rio de Janeiro 28895-532, Brazil

**Keywords:** androgen receptor signaling, osteopontin, splice variant, OPNc

## Abstract

Androgen receptor (AR) signaling is a key pathway modulating prostate cancer (PCa) progression. Several steps in this pathway have been investigated in order to propose novel treatment strategies for advanced PCa. Total osteopontin (OPN) has been described as a biomarker for PCa, in addition to its role in activating the progression of this tumor. Based on the known effects of the OPNc splice variant on PCa progression, the present study investigated whether this isoform can also modulate AR signaling. In order to test this, an *in vitro* model was used in which LNCaP cells were cultured in the presence of conditioned medium (CM) secreted by PCa cells overexpressing OPNc (OPNc-CM). The activation of AR signaling was evaluated by measuring the expression levels of AR-responsive genes (ARGs) using quantitative polymerase chain reaction and specific oligonucleotides. The data demonstrated that all nine tested ARGs (*Fgf8*, *TMPRSS2*, *Greb1*, *Cdk2*, *Ndrg1*, *Cdk1*, *Pmepa1*, *Psa* and *Ar*) are significantly upregulated in response to OPNc-CM compared with LNCaP cells cultured in CM secreted by control cells transfected with empty expression vector. The specific involvement of OPNc was demonstrated by depleting OPNc from OPNc-CM using an anti-OPNc neutralizing antibody. In addition, by using a phosphoinositide 3-kinase (PI3K)-specific inhibitor and AR antagonists, such as flutamide and bicalutamide, it was also observed that upregulation of ARGs in response to OPNc-CM involves PI3K signaling and depends on the AR. In conclusion, these data indicated that OPNc is able to activate AR signaling through the PI3K pathway and the AR. These data further corroborate our previous data, revealing the OPNc splice variant to be a key molecule that is able to modulate key signaling pathways involved in PCa progression.

## Introduction

Osteopontin (OPN) is a matricellular glyco-phosphoprotein that is overexpressed in several tumor types (1). In prostate cancer (PCa) samples, OPN is upregulated and mediates tumor progression (2,3). Moreover, high circulating OPN levels have been found in PCa patients, thus highlighting a putative biomarker role for OPN in PCa. The OPN transcript can occur as three distinct splice variants, OPNa, OPNb and OPNc (4), with tissue- and tumor-specific roles (5). Nonetheless, the majority of studies have explored the function of the full-length OPN in PCa (6–9). Notably, we have previously demonstrated that the overexpression of OPNc, and to a lesser extent OPNb, promotes PCa progression (10). Indeed, OPNc upregulation in PCa cells, which correlates to the Gleason score, induces PCa cell proliferation, migration, invasion, metastasis and tumor formation *in vivo*, mainly mediated by the PI3K pathway. Overall, our studies have shed light on the potential use of OPNc as a diagnostic and prognostic biomarker for PCa (11).

Disruption of androgen-mediated differentiation has been strongly linked to PCa development. Androgens, which bind to androgen receptors (AR) to elicit their cellular effects, are the primary sex hormones required for normal development, maintenance and differentiation of the male phenotype. AR-regulated genomic events modulate cell differentiation and the development of tissues and organs (12). Furthermore, AR signaling has been indicated as a key step for PCa progression (13), in which circumstance there is crosstalk with multifunctional growth factor signaling pathways, such as EGF, FGF, IGF, TGF-β and VEGF (14), as well as with the PI3K/Akt/mTOR pathway (15). In addition, it has been proposed that castration-resistant PCa evolution may be the result of increased growth factor signaling activity associated with intratumoral testosterone production (16,17).

Despite the key importance of AR signaling on PCa progression, to date, there have been no studies demonstrating the putative correlations among total OPN or its splice variants and AR signaling. The exception to this is a single study, which reported that upregulated *Fgf-8*, an androgen target gene, induces total OPN expression in PCa cells (18).

The present study describes an *in vitro* model in which LNCaP androgen-responsive PCa cells are used to investigate the differential modulation of AR target genes by the conditioned medium (CM) secreted by PCa cells overexpressing OPNc (OPNc-CM).

## Materials and methods

### Cell culture

The LNCaP cell line was used as an *in vitro* model to examine whether the AR pathway modulated by OPNc in PCa cells. The LNCaP cell line was obtained from the American Type Culture Collection (Rockville, MD, USA) and maintained in RPMI-1640 medium (Sigma-Aldrich, St. Louis, MO, USA) supplemented with 10% (v/v) heat-inactivated fetal bovine serum (FBS; Invitrogen Life Technologies, Carlsbad, CA, USA), in the presence of 100 U/ml penicillin and 100 μg/ml streptomycin, at 37°C in a 5% CO_2_ humidified incubator. The cells were maintained in medium containing charcoal/dextran-stripped FBS (CCS; Invitrogen Life Technologies) for three days prior to assaying the modulatory effect of OPNc-overexpressing secreted CM on LNCaP cell AR signaling.

### OPNc plasmid constructs, transfection and preparation of CM

In order to prepare the OPNc-CM, OPNc overexpression vector, which was kindly donated by Dr George Weber (Cincinnati University, Cincinnati, OH, USA), was used for transfections into a PC-3 prostate tumor cell line. The transfections were performed using Lipofectamine™ 2000, following the manufacturer’s instructions (Invitrogen Life Technologies). Cell clones stably overexpressing OPNc and empty vector (EV) control clones were selected using G418 at 800 μg/ml. Data from our previous study demonstrated that PC-3 stably transfected cells contain high levels of the protein and RNA transcript of OPNc in relation to their endogenous levels in EV-transfected cells (10). In order to prepare the CM secreted from OPNc-overexpressing cells and those expressing EV, cell number was normalized by plating PC-3 cells at the same cell density (5×10^5^ cells/well). Subsequent to reaching 80% cell confluence, the cells were washed twice with phosphate-buffered saline and cultured with RPMI in serum-free conditions for 48 h. Collected CM was clarified by centrifugation at 1,200 × g for 5 min. All assays were performed using freshly prepared CM. CM produced by OPNc-overexpressing cells or those transfected with EV controls, termed OPNc-CM and EV-CM, respectively, were used for the LNCaP assays over 24 h.

### LNCaP assays and AR signaling analysis

The LNCaP cells were plated in 2.0 ml RPMI without antibiotics at a density of 1.5×10^5^ cells/well, and maintained in medium containing CCS (Invitrogen) for three days prior to treatment with OPNc-CM or EV-CM, containing either anti-OPNc neutralizing antibody, LY294002, flutamide and bicalutamide, individually or in distinct combinations. LY294002, a PI3K inhibitor, was obtained from Cell Signaling Technology Inc. (Danvers, MA, USA). The LNCaP cells were cultured and treated with 50 mM LY294002. For OPNc depletion in OPNc-CM, 4 mg/ml of an anti-OPNc antibody (Gallus Immunotech, Cary, NC, USA) was used. This antibody was produced by immunizing a chicken with a peptide representing the splice junction of OPNc (Ac-SEEKQNAVSCCOOH). Specific binding to OPNc has been demonstrated by the manufacturers (Gallus Immunotech), and we have previously demonstrated that this antibody blocks PC3 cell proliferation in response to OPNc-overexpression (10). OPNc-CM was pre-incubated with anti-OPNc antibody for 2 h prior to LNCaP cell treatment. Assays using AR antagonists were performed using OPNc-CM containing 100 nM flutamide or 10 μM bicalutamide (Sigma-Aldrich). The LNCaP cells were allowed to grow for 24 h following the treatments, and then harvested for the analysis of gene expression. The mRNA expression levels of the androgen-responsive genes (ARGs), *Ar*, *Psa*, *Tmprss2*, *Ndrg1*, *Greb1*, *Fgf8*, *Cdk1*, *Cdk2* and *Pmepa1*, were analyzed using quantitative reverse transcription polymerase chain reaction (RT-qPCR).

### Total RNA isolation and RT

LNCaP total RNA was purified using the RNeasy Mini kit, using RNase-free DNase (Qiagen, Hilden, Germany) following the RNA purification process. Total RNA (1 μg) was reverse-transcribed into cDNA using a Superscript II First-Strand Synthesis System for RT-PCR (Invitrogen). The resulting cDNA was quantified using a NanoDrop™ 1000 spectrophotometer (Thermo Scientific, Waltham, MA, USA).

### qPCR

qPCR was performed using a CFX96 Real-Time System with a C1000 Thermal Cycler (Bio-Rad, Hercules, CA, USA), and SYBR Green (Applied Biosystems, Foster City, CA, USA), according to the manufacturer’s instructions. The oligonucleotide primers used for the qPCR are listed in Table I. The expression levels of *Ar*, *Psa*, *Tmprss2*, *Ndrg1*, *Greb1*, *Fgf8*, *Cdk1*, *Cdk2* and *Pmepa1* were normalized based on the reference gene (18S rRNA), using the ΔΔCT relative quantification method. Conditions for PCR amplification were as follows: 50°C for 2 min and 94°C for 5 min, followed by 40 cycles at 94°C for 30 sec, 50°C for 30 sec and 72°C for 45 sec, and a final extension at 72°C for 15 min. To evaluate the specificity of the PCR products, a melting curve analysis was performed after each reaction.

### Statistical analyses

All the statistical analyses were performed using SPSS software version 18.0 (SPSS, Inc., Chicago, IL, USA). Data were analyzed by comparison using a two-tailed t-test, and P<0.05 was considered to indicate a statistically significant difference.

## Results and Discussion

An improved understanding of the molecular mechanisms triggering AR signaling in PCa cells strongly relies on dissecting gene products and factors able to stimulate this hormonal pathway. Moreover, novel therapeutic strategies against PCa, mainly in recurrent disease, may attempt to target AR pathway elements or its stimulatory mechanisms (17). We previously demonstrated that the OPNc splicing isoform stimulates several PCa tumor progression features, including cell proliferation, migration, invasion, metastatic potential and tumor formation *in vivo* (10). Although other studies have shown that total OPN stimulates LNCaP cell proliferation in the presence of EGF (19), to date, information about the effect of distinct OPN splice variants on AR pathway modulation in PCa is lacking. We have previously shown that the majority of the OPNc-mediated PCa features are specifically modulated by OPNc-CM (10). In addition, we have demonstrated that OPNc-CM secreted by PC3 cells differentially modulates several cancer-related genes (20).

**Figure 1:**
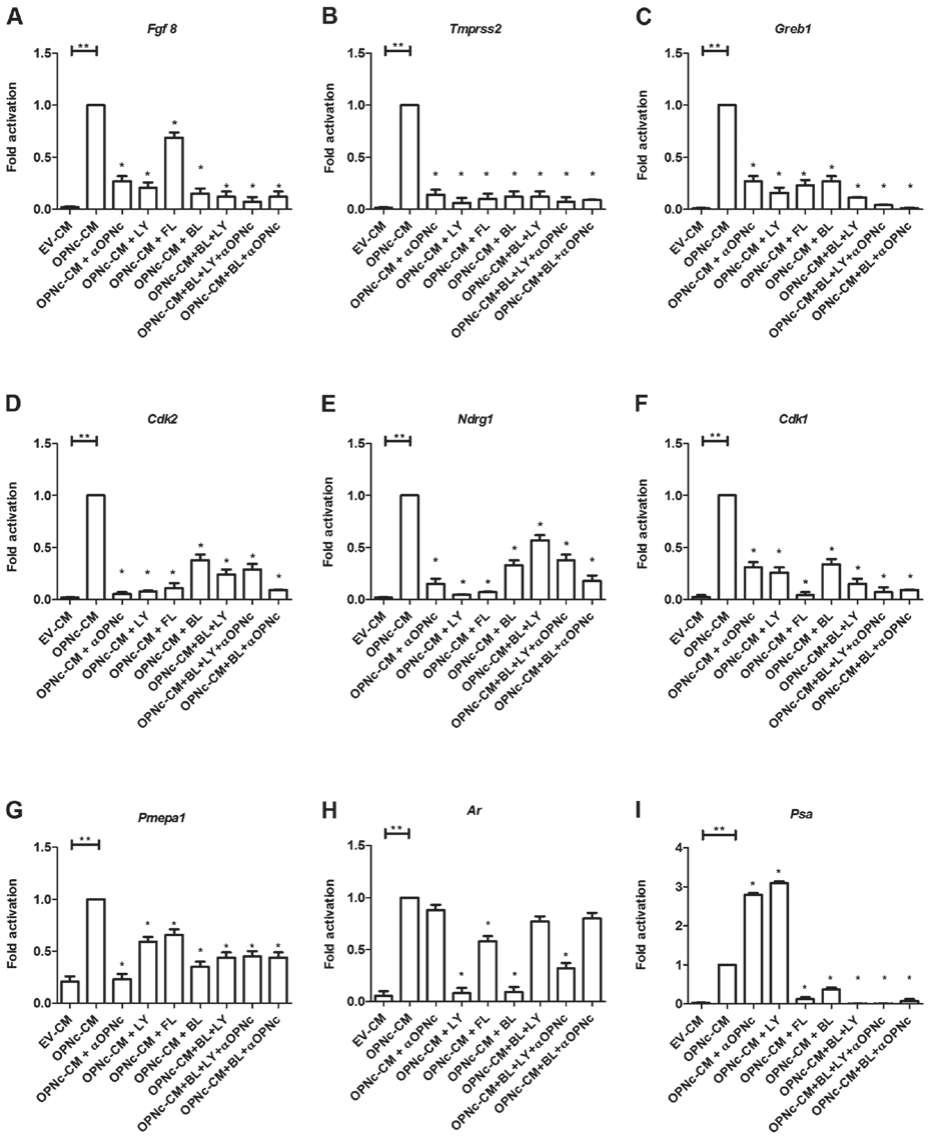
Conditioned medium (CM) secreted by PCa cells overexpressing OPNc (OPNc-CM) significantly activates the expression of AR responsive genes in LNCaP cells. Graphs showing relative RNA quantification of (A) *Fgf8*, (B) *Tmprss2*, (C) *Greb1*, (D) *Cdk2*, (E) *Ndrg1*, (F) *Cdk1*, (G) *Pmepa1*, (H) *Ar* and (I) *Psa,* in LNCaP cells cultivated with OPNc-CM compared with cells cultivated with the empty-vector CM (EV-CM), as described in the Materials and methods section. In order to test OPNc-specific effects, anti-OPNc polyclonal neutralizing antibody (α-OPNc), flutamide (FL), bicalutamide (BL) and PI3K inhibitor (LY), were used. 18S RNA was used as a constitutive gene in all these assays. Data are represented as the mean ± standard deviation. All experiments were biological replicates repeated a minimum of three times. ^*^P<0.0001 vs. OPNc-CM cultivated cells. ^**^P<0.0001 vs. EV-CM cultivated cells.

The present study used OPNc-CM to investigate the modulation of AR signaling, by evaluating the expression patterns of ARGs in LNCaP androgen-responsive cells. OPNc-CM, but not EV-CM, significantly increased the expression of all nine ARGs tested (Fig. 1). These data support our previous findings that OPNc stimulates several aspects of PCa progression (10), possibly through an AR signaling-mediated pathway. All tested ARGs have been described with regard to the modulation of PCa growth and progression (21); the *Fgf8* (22), *Cdk1* (23), *Cdk2* (24) and *Greb11* (25) gene products are classically involved in prostate cell growth and proliferation. Although *Psa* has been classically described as an oncogene in PCa, promoting tumor progression and metastasis, its function as a tumor suppressor molecule has been also documented (23). *NDRG1-ERG* fusions, which encode a chimeric protein, are also regulated by androgens and correspond to one of the recurrent erythroblast transformation-specific rearrangements observed in PCa. Presumably, *Ndrg1* promotes angiogenesis, metastasis and drug resistance (26). *Tmprss2*, which is another component of typical androgen-regulated PCa translocations, is expressed in PCa cells and contributes to prostate tumorigenesis (27,28). By contrast, the *Pmepa1* gene, although a direct target of the AR, has been described as negatively regulating prostate epithelium cell growth, in addition to the AR protein levels in a range of cell culture models (29,30).

In order to investigate the specificity of the effect of OPNc on the upregulation of tested ARGs, LNCaP cells were cultured with OPNc-CM pre-treated with an anti-OPNc polyclonal neutralizing antibody. The expression of seven out of nine of the ARGs (*Fgf8*, *Tmprss2*, *Greb1*, *Cdk2*, *Ndrg1*, *Cdk1* and *Pmepa1*) was not increased when OPNc activity was abrogated by this anti-OPNc antibody. These data indicated the specific effect of OPNc on inducing the upregulation of these seven ARGs in response to OPNc-CM (Fig. 1A–G). Conversely, *AR* transcript upregulation was sustained regardless of the OPNc activity, therefore suggesting that secreted factors contained in OPNc-CM, other than OPNc, could mainly contribute to *AR* transcript expression in PCa cells. Lastly, abrogation of OPNc activity further stimulated *PSA* expression in the LNCaP cells (Fig. 1I), thus indicating that secreted OPNc is a partial inhibitor of *PSA* expression in the PCa cell line. These results indicate that direct or indirect OPNc-mediated mechanisms, either in the OPNc-CM or within the LNCaP cells, could suppress PSA transcript expression. It has previously been demonstrated that during PCa progression, intracellular PSA levels are lower in the malignant rather than the normal prostatic epithelium, being further reduced in poorly-differentiated tumors, despite the high serum levels of PSA detected in patients with PCa. Moreover, PSA can function as a tumor suppressor by inhibiting tumor angiogenesis in PCa cells (31). In fact, regardless of the widespread use of PSA as a PCa marker, it has been established that the modulation of PSA expression and the proliferation of PCa cells are independently regulated during the development and progression of the disease. It is notable that multiple factors have been indicated to be involved in the transcriptional transactivation of PSA; mainly AR, but also various growth factors and extracellular matrix proteins (32,33). We hypothesize that this could be the case for OPNc. Hence, it is possible that OPNc or growth factors secreted in response to the overexpression of this splice variant could negatively modulate PSA expression in LNCaP cells, using androgen-dependent and/or independent mechanisms, in order to favor PCa progression.

The precise mechanisms responsible for the aberrant AR expression in PCa remain elusive (34). The data from our studies has shed light on the complexity of the phenomenon, indicating that not only secreted OPNc, but also other secreted factors in response to OPNc overexpression, positively modulate AR signaling in LNCaP cells. In this context, other studies have also described the stimulatory effects of extracellular and intracellular signaling molecules on AR-mediated transcription, such as heparin-binding EGF-like growth factor, activin A, Smad2 and angiotensin II receptor type 1 (35). Further studies should be performed to determine the factors produced in response to OPNc overexpression, as well as the molecular mechanisms these molecules can induce in order to modulate AR-mediated signaling in PCa cells.

Our previous studies recently demonstrated that OPNc modulates the PI3K pathway (10), as well as other key cancer pathways (20). As a consequence, we hypothesize that OPNc modulates the signaling of several growth factors in PCa cells, which in turn, induce ARG expression, exactly as previously reported for other oncoproteins, such as PLK1 (36), ETV1 (37) and ELK1 (38). It is possible that OPNc can also stimulate LNCaP endogenous androgen synthesis, similar to the role of interleukin-6 on the *de novo* synthesis of intracrine androgens (39). Indeed, our previous data have clearly shown that PI3K mediates several OPNc tumor progression features in PC3 cells overexpressing this splice variant (10). In the present study, it was demonstrated that the PI3K pathway was also involved with OPNc-mediated ARG expression in the LNCaP cells, with the exception of PSA, whose expression was not abrogated by the PI3K inhibitor LY294002 (Fig. 1). Altogether, these results shed light on the PI3K pathway as a key mechanism for OPNc-mediated effects on LNCaP cells (10,15). Upon further consideration of the crosstalk between the PI3K and AR pathways, we postulate that OPNc can modulate each pathway (15). In this context, we propose that the observed PSA upregulation, regardless of the PI3K pathway activity status, could be mainly due to the depletion of OPNc-activated signals as an inhibitor of PSA expression, similar to the experimental conditions in which the anti-OPNc antibody was used to abrogate OPNc activity in OPNc-CM. We finally propose that the tumor progression features modulated by OPNc in PCa cells may be derived, at least in part, from the PI3K-activated upregulation of ARGs.

Significantly, the present study demonstrated that AR mediates the OPNc-CM-activated upregulation of all ARGs tested, as the observed phenomena were significantly reversed when the LNCaP cells were pre-treated with the AR antagonists flutamide and bicalutamide (Fig. 1). The expression of the OPNc-CM-activated ARGs was also assessed in the LNCaP cells in the presence of LY294002 or bicalutamide. With the exception of AR, the expression of all other ARGs was significantly reduced. Also, the combined treatment with LY294002 and bicalutamide promoted a stronger effect on the inhibition of the OPNc-CM-mediated upregulation of the ARGs in the LNCaP cells, as compared to the individual bicalutamide or LY294002 treatments. Therefore, these data could provide support to strategies that could target the PI3K and AR signaling pathways as an efficient approach to inhibit PCa progression, as discussed in the study by Bitting and Armstrong (15).

In conclusion, to the best of our knowledge, the present study is the first to use PCa cells overexpressing OPNc and report that OPNc and/or other secreted factors are key elements modulating the AR signaling pathway. Briefly, the data indicate that OPNc-CM induces the expression of ARGs in LNCaP cells mainly through the activation of the PI3K and AR pathways; the latter being activated either by secreted OPNc-CM or LNCaP endogenously-produced AR ligands. This reinforces that these signaling pathways have key roles in mediating OPNc-related tumor progression features in PCa. Further studies should investigate the specific molecular pathways by which OPNc modulates the AR signaling and the secreted factors expressed in response to OPNc overexpression that could also contribute to AR signaling activation. Based on these results, therapeutic strategies trying to target OPNc and its downstream PI3K and AR pathways should also be considered in order to negatively modulate PCa progression.

## Figures and Tables

**Table I:** Oligonucleotide primers used for analysis of RT-qPCR expression of androgen receptor-responsive genes.

Gene	Accession number	Primer (5′-3′)
*Pmepa1*	NC_000020.11	F: CATGATCCCCGAG CTGCT R: TGATCTGAACAAACTCCAGCTCC
*TMPRSS2*	NC_000021.9	F: CTGGTGGCTGATAGGGGATA R: GGACAAGGGGTTAGGGAGAG
*Ndrg1*	NC_000008.11	F: CGAGACTTTACATGGCTCTG R: GCATTGATGAACAGGTGCAG
*Greb1*	NC_000002.12	F: AAGGAGGGCTGGAAACAAAT R: CATTGTGGCCATTGTCATCT
*Psa*	NC_000019.10	F: TGCATCAGGAACAAAAGCGTGA R: CCTGAGGCGTAGCAGGTGGTCCCCAG
*Ar*	NC_000023.11	F: GGTGAG CAGAGTGCCCTATC R: GAAGACCTTGCAGCTTCCAC
*Fgf8*	NC_000010.11	F: CAACTCTACAGCCGCACCAGC R: TGCTCTTGGCGATCAGCTTC
*Cdk1*	NC_000010.11	F: AAGTGAAGAGGAAGGGGTTCC R: CCAAAAGCTCTGGCAAGGCC
*Cdk2*	NC_000012.12	F: ATGGGTGTAAGTACGAACAGG R: TTCTGCCATTCTCATCGG
*18S*	NT_167214.1	F: AACCCGTTGAACCCCATT R: CCATCCAATCGGTAGTAGCG

F, forward; R, reverse.
